# Estimating dementia prevalence using remote diagnoses and algorithmic modelling: a population-based study of a rural region in South Africa

**DOI:** 10.1016/S2214-109X(24)00325-5

**Published:** 2024-12

**Authors:** Meagan T Farrell, Darina T Bassil, Muqi Guo, M Maria Glymour, Ryan G Wagner, Stephen Tollman, Kenneth M Langa, Adam M Brickman, Jennifer J Manly, Lisa F Berkman

**Affiliations:** Harvard Center for Population and Development Studies, Harvard University, Cambridge, MA, USA (M T Farrell PhD, D T Bassil PhD, M Guo MS, Prof L F Berkman PhD); Department of Global Health and Population (M Guo) and Department of Social and Behavioral Sciences (Prof L F Berkman), Harvard T H Chan School of Public Health, Harvard University, Cambridge, MA, USA; Department of Epidemiology, Boston University School of Public Health, Boston, MA, USA (Prof M M Glymour PhD); MRC/Wits Rural Public Health and Health Transitions Research Unit (Agincourt), School of Public Health, Faculty of Health Sciences, University of the Witwatersrand, Johannesburg, South Africa (R G Wagner PhD, Prof S Tollman PhD, Prof L F Berkman); INDEPTH Network, Accra, Ghana (Prof S Tollman); Institute for Healthcare Policy and Innovation and Institute for Social Research, University of Michigan, Ann Arbor, MI, USA (Prof K M Langa MD); Department of Internal Medicine, University of Michigan Medical School, Ann Arbor, MI, USA (Prof K M Langa); Veterans Affairs Center for Clinical Management Research, Ann Arbor, MI, USA (Prof K M Langa); Taub Institute for Research on Alzheimer’s Disease and the Aging Brain, Gertrude H Sergievsky Center, and Department of Neurology, Vagelos College of Physicians and Surgeons, Columbia University, New York, NY, USA (Prof A M Brickman PhD, Prof J J Manly PhD)

## Abstract

**Background:**

Dementia is a leading cause of global death and disability. High-quality data describing dementia prevalence and burden remain scarce in sub-Saharan Africa. Health and Aging in Africa: A Longitudinal Study in South Africa (HAALSI) fills evidence gaps with longitudinal data on cognition, biomarkers, and everyday function in a population-based cohort of Black South Africans, aged 40 years and older, in a rural subdistrict. This study uses consensus diagnoses and prediction algorithms to estimate dementia prevalence.

**Methods:**

Data were from eligible HAALSI Wave 2 respondents aged 50 years or older (n=3662) and were collected between September, 2019, and January, 2020. An enriched sub-cohort (ie, including a high proportion of individuals with cognitive impairment; n=632) completed a battery of rigorous neuropsychological and clinical assessments and received expert classification of cognitively unimpaired, mild cognitive impairment, or dementia. Logistic regression was used to predict dementia status within the sub-cohort using predictor variables from the parent HAALSI wave. Coefficients were applied to the parent cohort to obtain dementia probability scores and calculate dementia prevalence. Optimal probability cut points to classify individual cases were selected for each model.

**Findings:**

When the sub-cohort was reweighted to reflect the full HAALSI population, the estimated prevalence of dementia was 18% (95% CI 15–22), with steep age gradients. Four models of increasing complexity showed good discrimination between dementia and non-dementia (area under receiver operating characteristic curves 0·78–0·84; classification accuracy 74–81%). Model-based dementia prevalence estimates aligned closely with weighted prevalence; model performance was consistent in cross-validated datasets.

**Interpretation:**

HAALSI is among the first studies to use algorithmic methods to describe dementia prevalence in a population-based sample in South Africa. These efforts could provide a foundation to expand understanding of dementia epidemiology in a region of the world experiencing rapid population ageing.

**Funding:**

National Institute on Aging.

## Introduction

With global population ageing, an increasing number of people are living with dementia,^1,2^ posing an unprecedented challenge for health and social infrastructures around the world. Although age-specific prevalence and incidence of dementia might be declining in some world regions,^3,4^ dementia remains a leading cause of global death and disability owing to the sheer volume of individuals living to older ages. By 2050, more than 70% of people living with dementia will reside in low-income and middle-income countries.^5^ A true understanding of the epidemiology of dementia in low-income and middle-income countries, especially in sub-Saharan Africa, is limited by the scarcity of population-based dementia data from these countries. In this study, we aimed to estimate the prevalence of dementia in a rural area of South Africa using data from The Health and Aging in Africa: A Longitudinal Study of an INDEPTH Community in South Africa (HAALSI) study.^6^

In sub-Saharan Africa, older adults (ie, those aged >65 years) will make up an increasing proportion of the population due to increasing life expectancy and reductions in fertility.^7,8^ Risk factors for dementia, including limited educational opportunity, impaired sensory function, and poorly controlled cardiovascular conditions, are common in the region.^9–11^ Published dementia prevalence estimates vary dramatically across sub-Saharan African studies, likely due to methodological variabilty.^10^ Population-based studies incorporating longitudinal measurements of cognition and everyday function are needed to characterise the burden of dementia in sub-Saharan Africa.

Estimating dementia remains challenging at the population level, particularly in novel research settings in which neuropsychological measures have not been fully validated and normative data have not been developed. For longitudinal studies, diagnostic adjudication procedures are the gold standard for ascertaining dementia status. This approach includes administration of a comprehensive battery of neuropsychological tests, medical examinations, and informant interviews; data are then reviewed by an expert panel of clinicians who assign a final diagnostic outcome via consensus. Although diagnostic adjudication provides the most rigorous approach, it is resource-intensive and difficult to implement in larger samples. Conversely, some population-based studies use cognitive screening tests to define dementia status, which can result in high rates of misclassification.^12^

To circumvent these challenges, HAALSI uses a combination of diagnostic adjudication procedures and data-driven algorithms to improve precision of dementia ascertainment. Since 2014, HAALSI has collected broad measures of cognitive and everyday functioning, physical and mental health, social wellbeing, and socioeconomic factors to characterise trajectories of physical and cognitive ageing in the population.^6^ The HAALSI Dementia-Harmonized Cognitive Assessment Protocol (HAALSI-HCAP) is a sub-study nested within the larger HAALSI programme. The HAALSI-HCAP sub-cohort, which includes a high proportion of participants who are cognitively impaired, receives additional neuropsychological, functional, and biomarker assessments to facilitate the measurement of dementia in the cohort.^13^ HAALSI is part of a global network of harmonised ageing studies, including the United States Health and Retirement Study^14^ and international partners.

The objectives of this paper are to describe dementia prevalence in a rural South African HAALSI cohort, evaluate performance of various algorithms for dementia ascertainment, and provide a foundation for future research on dementia incidence in HAALSI, with implications for dementia research in the sub-Saharan African region.

## Methods

### Study setting and design

HAALSI is a population-based, prospective, cohort study of adults aged 40 years and older in the Agincourt sub-district of Mpumalanga Province, South Africa.^15^ Agincourt remains challenged by converging epidemics of communicable and non-communicable diseases and limited health-care infrastructure.^15^ HAALSI cohort members report low educational attainment and high lifelong unemployment associated with the legacies of colonialism and apartheid. These contextual factors are important to consider when interpreting rates of cognitive impairment and dementia in the cohort. HAALSI is nested within a Health and Demographic Surveillance System research site,^15^ and studies have shown marked demographic similarity across rural communities in South Africa and the sub-Saharan African region.^16^

**Figure 1:**
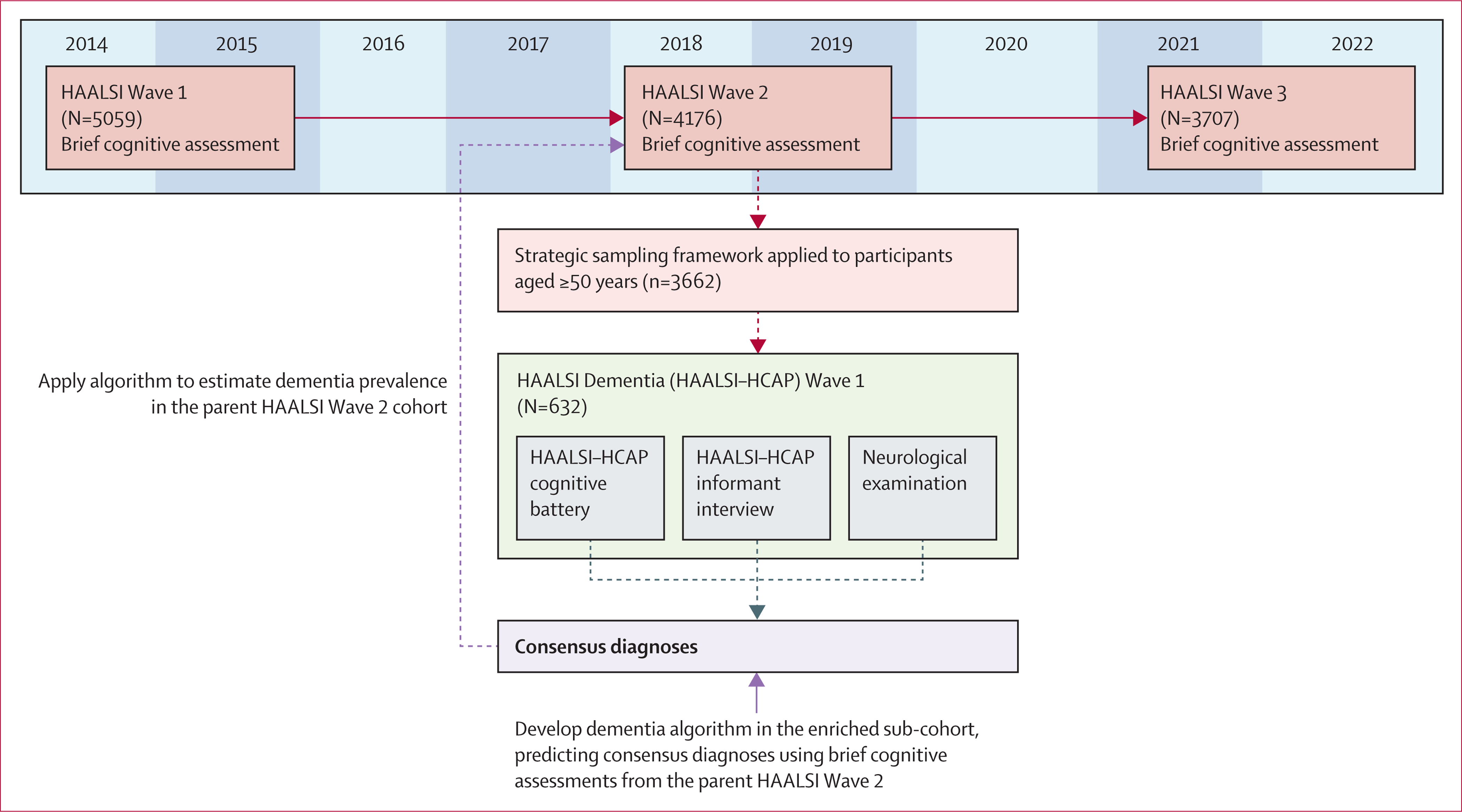
Study design of HAALSI and HAALSI–HCAP HAALSI=Health and Aging in Africa: A Longitudinal Study in South Africa. HAALSI–HCAP=HAALSI Dementia–Harmonized Cognitive Assessment Protocol.

The baseline HAALSI wave was conducted in 2014–15 (n=5059), with two follow-up waves completed in 2018–19 and 2021–22 (figure 1). The parent HAALSI study implements a broad survey, including brief cognitive assessments and activities of daily living (ADLs). For cases in which respondents were unable to complete the interview due to illness or cognitive impairment, a proxy assessment was conducted with a close contact who was asked to evaluate respondents’ cognitive and physical health.

Modelled off the United States Health and Retirement Study Aging, Demographics, and Memory Study,^17^ the HAALSI–HCAP study used a wave-level planned missingness design,^18^ in which only a segment of the HAALSI sample was selected to complete the inter-wave dementia diagnostic assessment (figure 1). A strategic sub-sample of 632 respondents aged 50 years and older was selected from the second wave of the parent HAALSI study.^13^ Approximately 6 months after HAALSI Wave 2, HAALSI–HCAP respondents completed the HCAP battery and were assigned diagnostic outcomes of cognitively unimpaired, mild cognitive impairment, or dementia by an expert clinical panel.^19^ After, known relationships between dementia diagnoses and measures available at HAALSI Wave 2 were used to extrapolate dementia prevalence to the full sample. This two-stage approach allowed for a more accurate estimation of dementia prevalence than using a single cognitive score.

Ethical approval was obtained through Harvard University, University of the Witwatersrand, and the Mpumalanga Provincial Research and Ethics Committee. Per local ethical standards, informed consent was obtained and documented through written consent (for literate participants), verbal consent with witness (for illiterate participants), or proxy consent (for participants with severe cognitive impairment).

### Sampling

Respondents aged 50 years and older in HAALSI Wave 2 were eligible for selection into HAALSI–HCAP. Compared with most dementia epidemiology studies, HAALSI intentionally enrolled a younger sample to explore pre-dementia pathways that might unfold in middle age. A strategic sampling distribution was developed to enhance precision in estimating dementia prevalence. A full description of the sampling procedures is provided in appendix 1 (p 2). In brief, HAALSI respondents were stratified by their cognitive performance on the HAALSI Wave 2 screening tests. Respondents with low cognitive scores were oversampled to facilitate development of prediction models. Sampling weights were constructed as the inverse probability of selection into HAALSI–HCAP based on one’s cognitive performance stratum.

### Measures

Measures of cognition and everyday function administered during parent HAALSI waves were used to predict dementia diagnostic outcomes in the HAALSI–HCAP sub-cohort. Most measures were harmonised with the United States Health and Retirement Study, with cultural adaptations. Details on these assessments are published elsewhere.^20,21^

The HAALSI cognitive battery comprised of immediate and delayed word recall, orientation, subjective memory ratings, a shapes-based trail-making test, picture naming, verbal fluency, incidental picture memory, listing days of the week forward and backward, and adaptive number series. Self-reported ADL limitations included walking, bathing, eating, toileting, and dressing. Limitations in eight instrumental ADLs were assessed, including meal preparation, grocery shopping, taking medications, managing money, using telephones, performing household chores, looking up information, and travelling. Algorithms are intended to be broadly applied, so measures with substantial (ie, ≥5%) missingness, including trail making, picture naming, number series, and incidental memory, were excluded from core algorithms.

Proxy interviews for individuals unable to complete the interview included assessment of respondents’ current cognitive ability and changes in cognitive ability over the past 2 years, as well as limitations in ADLs and instrumental ADLs.^6^

In HAALSI–HCAP, consensus-based classifications of dementia status were used as the gold-standard outcome for development of algorithms.^19^ For consensus diagnoses, data from HAALSI–HCAP neuropsychological battery, informant interviews, and neurological examination were accessed by an expert panel of South African and American clinicians via an online portal.^19^ Each case was reviewed by three independent raters who assigned a diagnostic outcome of cognitively unimpaired, mild cognitive impairment, or dementia. Unanimous agreement among three raters was required before final outcomes were assigned.^19^ Cases without universal agreement on diagnostic category were discussed at bi-weekly adjudication conferences until consensus was reached.

Diagnosis of dementia followed the National Institute on Aging-Alzheimer’s Associations 2011 criteria for dementia,^22^ requiring evidence for cognitive impairment in at least two cognitive domains that had (1) declined over time, (2) interfered with social, occupational, or everyday function, and (3) not been explained by delirium or psychiatric disorder. Respondents showing cognitive impairment (through informant report or cognitive testing) without functional interference were categorised as mild cognitive impairment. For algorithm development, we focused on the presence of dementia and defined those with normal cognitive function and mild cognitive impairment as no dementia.

### Statistical analysis

We used two methods to estimate dementia prevalence. First, we applied sampling weights to the observed dementia outcomes obtained in HAALSI–HCAP to extrapolate prevalence among all HAALSI respondents aged 50 years and older. In the second method, logistic regression models were developed using variables from the parent HAALSI Wave 2 to predict consensus diagnoses at HAALSI–HCAP Wave 1 (see appendix 1 p 5 for model equations). Model 1 included brief cognitive measures and ADLs. Predictors were then iteratively added to models, examining model performance at each step to determine whether additional variables improved model fit. Model 2 added age main effects and interactions; model 3 added more cognitive measures, instrumental ADLs, sex, and education level. In model 4, we evaluated interactions of age, sex, and education with cognitive and functional measures using least absolute shrinkage and selection operator regression to avoid overfitting. Variables included in each model are shown in appendix 1 (p 7).

Subsequently, we applied model coefficients to the HAALSI Wave 2 dataset and generated dementia probability scores for each eligible respondent in the parent HAALSI cohort. For each model, the prevalence of dementia was calculated as the average probability score, both overall and across age groups.

Comparisons were made across models based on area under the receiver operating characteristic (AUROC) curves. We then identified an optimal probability cut point for each model that would best distinguish between dementia and no dementia at the individual level. We examined the distribution of probability scores and evaluated cut points in terms of sensitivity, specificity, and accuracy for classifying dementia. At each cut point, we calculated Youden’s J statistic (ie, sensitivity + specificity – 1). Given the purpose of this cut point was to generate accurate estimates of dementia prevalence, cut points were selected to maximise the correct classification rate (accuracy). Specifically, an optimal cut point was selected for each model to enable the highest overall classification accuracy while maintaining 70% sensitivity.

To evaluate reliability of algorithms and cut points, we performed five-fold cross-validation. We then calculated model performance metrics for the cross-validated datasets and compared them with performance metrics observed in the full dataset. Finally, we used the bootstrap percentile method with 100 iterations to provide CIs for the performance metrics of algorithms (appendix 1 p 9). Detailed descriptions of cross-validation and bootstrapping procedures are reported in appendix 1 (p 5). Performance metrics of the algorithms and cut points were compared across gender, age, and education level subgroups.

Respondents who required proxies to complete HAALSI Wave 2 were not included in algorithm development owing to insufficient predictor data. However, results for models that incorporated proxy status as a dummy variable, whereby respondents with proxies were assigned 0 for all cognitive measures, are included in appendix 1 (p 10). Sensitivity analyses were also performed to test the performance of alternative models incorporating measures that were excluded from core models due to high missingness. All statistical analyses were conducted with Stata (version 16.1).

### Role of the funding source

The funder of the study had no role in study design, data collection, data analysis, data interpretation, or writing of the report.

## Results

Sample characteristics of self-respondents in the HAALSI Wave 2 cohort and the HAALSI–HCAP sub-cohort are shown in table 1. Due to intentional over-sampling of individuals with lower cognitive test scores, the HAALSI–HCAP sample was characterised by older age, a higher proportion of women, lower education levels, and individuals in lower income quartiles when compared with the parent sample (table 1).

After applying sampling weights to consensus-based diagnostic outcomes in the HAALSI–HCAP cohort, the estimated prevalence of dementia in HAALSI Wave 2 was 659 (18%) of 3662 when proxy respondents were included and 516 (15%) of 3440 when proxy respondents were excluded (table 2). Among self-respondents, dementia prevalence increased with age, ranging from 40 (4%) of 1010 participants aged 50–59 years to 179 (36%) of 498 participants aged 80 years and older.

**Figure 2:**
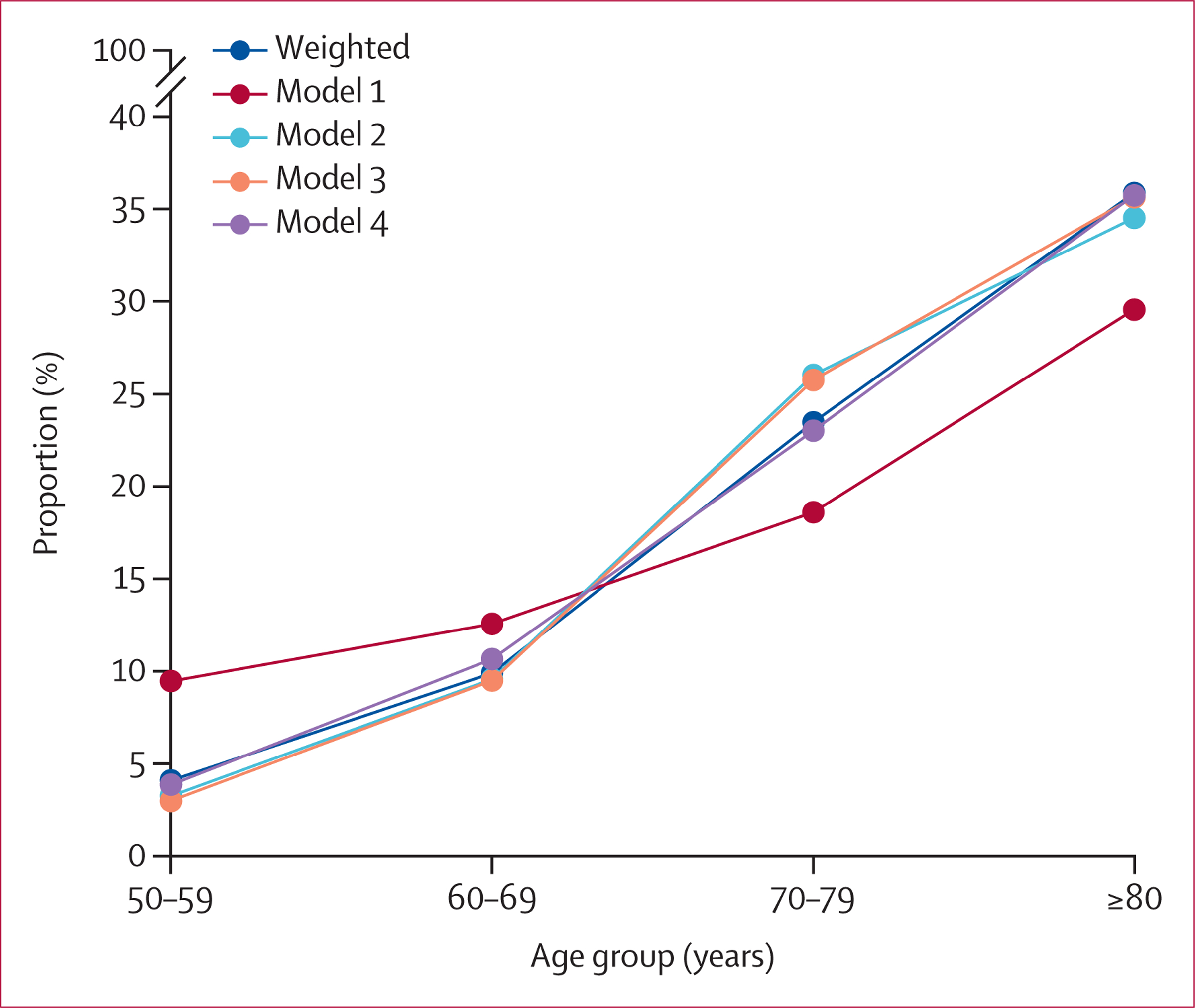
Predicted prevalence of dementia by age group across estimation methods Model 1: immediate and delayed word recall, orientation, self-rated memory, and activities of daily living. Model 2: model 1 predictors plus age and age × age ≥80 years. Model 3: model 2 predictors plus verbal fluency, days of the week, instrumental activities of daily living, education, and sex. Model 4: model 3 predictors plus interactions selected via least absolute shrinkage and selection operator.

Overall, dementia prevalence among self-respondents was highly similar across estimation methods (15·2–15·5%), with varying age-specific prevalence rates produced by different models (figure 2). The greatest variation occurred within the youngest and oldest age groups, with model 1 producing the highest dementia prevalence in the 50–59 years age group and the lowest prevalence in the ≥80 years age group. Model 1 is the only algorithm that does not include age as a predictor of dementia, and its prevalence estimates are least aligned with the weighted prevalence. Coefficient values for all models, showing that age was a strong predictor of dementia status across models 2–4, are shown in appendix 1 (p 8). Dementia prevalence according to each prediction method and broken down by age and education groups is shown in appendix 1 (p 6).

All models showed strong prediction capabilities, with AUROC values between 0·78 (model 1) and 0·84 (model 4; table 3). Table 3 presents performance metrics (sensitivity, specificity, accuracy, and Youden J statistic) for each model at selected cut points. Across models, accuracy rates ranged from 74·3% (model 1) to 80·7% (model 4), with sensitivities greater than 70·0% and specificities greater than 75·0%. Accuracy improved with the addition of predictors to the models. AUROC values were similar across demographic groups. However, selected probability cut points produced higher sensitivities but lower specificities in females, older respondents, and respondents without formal education. Demographic group differences were consistent across models.

Model performance metrics from cross-validated datasets, showing strong reliability of model performance over the five folds, are shown in appendix 1 (p 9). Models performed similarly when proxy status was added as a dummy variable (appendix 1 p 10) and when additional cognitive function measures were included (appendix 1 p 11).

## Discussion

These data provide crucial insights into dementia prevalence in a rural sub-district of South Africa, a country undergoing rapid demographic transition in an understudied world region. South Africa is a socially complex country with persisting extreme inequalities, and there is substantial value in generating rigorous epidemiological data in a marginalised rural population that reflects many rural populations of sub-Saharan Africa. Formal ascertainment of dementia status for research is time-consuming and costly, rendering it difficult to achieve in population-based studies. We offer an approach to generate more robust comparative evidence on dementia using harmonised cognitive assessments and clinical consensus diagnoses. We report a weighted overall dementia prevalence of 18%, characterised by steep age gradients. Across age groups, the HAALSI–HCAP dementia algorithms performed well against gold-standard consensus diagnoses, yielding estimates of dementia closely aligned with weighted estimates. Taken together, results support the use of algorithmic methods to facilitate reliable monitoring of dementia in longitudinal population-based studies, such as HAALSI.

Our study builds on the work of other research groups investigating dementia epidemiology in African samples, including the Ibadan Study of Aging,^23^ 10/66 pilot study in Nigeria,^24^ and other community-based studies in South Africa and throughout the region. There is considerable variability in dementia prevalence reported across studies in sub-Saharan Africa,^10^ which likely stems from differences in study settings, sampling practices, and procedures used to define dementia. Generally, hospital-based studies have reported the lowest dementia prevalence (as low as 2%), whereas community studies deploying comprehensive assessments (such as HAALSI) have reported rates of up to 20%.^10^ In direct comparison, two diagnostic approaches yielded drastically different dementia prevalence estimates in the same rural Tanzanian population, showing the complexity of pooling epidemiological data across studies.^25^ The 10/66 studies were among the first to implement rigorous dementia research in low-income and middle-income countries, with the 10/66 dementia algorithm showing good sensitivity for dementia detection in many countries.^26^ However, as noted by Prince and colleagues,^26^ the sample used to validate the 10/66 algorithm in Nigeria (n=76) was too small to fully analyse its performance. The HAALSI–HCAP algorithm addresses key challenges identified in the foundational 10/66 research, in that it was built and validated in a population-representative sample; relies on dementia outcomes determined via expert clinical consensus; and includes a more comprehensive assessment of cognitive domains, which could prove to be important for characterising dementia phenotypes.

One of the advantages of this approach is that the HAALSI–HCAP battery incorporates several cognitive assessments used in other African studies, including items from the Community Screening Interview for Dementia.^27^ The use of common items creates potential to statistically harmonise measures of cognitive impairment and dementia through latent variable methods,^28^ facilitating better understanding of both prevalence and shared risk factors for dementia across older populations in Africa.

After selecting optimal cut points for each model, the correct classification rates obtained with HAALSI–HCAP algorithms were generally on par with algorithms developed in similar studies.^29^ Despite high overall accuracy (greater than 80%), variability in the performance of probability cut points across demographic groups warrants further discussion. Model AUROC values were similar across demographic subgroups, suggesting that model predictors were strongly associated with dementia outcomes in all subgroups. Yet, cut points produced higher sensitivities and lower specificities in older, female, and less educated respondents. We are not the first to report differential performance of dementia algorithm cut points across demographic subgroups.^29^ The high accuracy of our models substantiates their use for the current study’s purpose, which is to estimate overall dementia prevalence in Agincourt, South Africa. However, future research focused on charactering demographic disparities in dementia prevalence, incidence, or risk factors might consider using continuous probability scores or subgroup-specific probability cut points to avoid misclassification bias.

Algorithmic methods will never perfectly replicate clinical diagnoses. For one, the panel reviewed comprehensive information not included in models, such as informant interviews and neurological examination findings. Although specific diagnostic criteria were applied, raters often use gestalt clinical opinion. Nuanced diagnostic processes, which require clinical expertise, are difficult to reproduce using purely quantitative modelling techniques. Dementia is an insidious condition. Diagnoses reflect progression past a clinically meaningful threshold rather than an abrupt change in function;^29^ as such, classifying respondents at a single timepoint is inherently challenging. Previous studies have shown that algorithmic methods for dementia ascertainment perform better at identifying prevalent dementia compared with new incident cases, when symptoms are less obvious.^29^ Discrete diagnostic categories for research might oversimplify the continuous underlying dimension of cognition in older age.

Accuracy of dementia algorithms will continue to be optimised. Additional longitudinal waves will enhance understanding of cognitively unimpaired test performance across age groups and levels of education. The true predictive power of these algorithms will only be fully understood by examining how well algorithmically assigned dementia status predicts future health states (ie, dementia and mortality) and biological markers of disease. Although we are primarily focused on describing the prevalence of all cause dementia, the breadth of the cognitive battery, clinical measures, and biomarkers in HAALSI could eventually reveal deeper insights into the patterning of dementia phenotypes. For example, HAALSI has a high rate of HIV positivity (23%). Although HIV prevalence is higher at the younger end of the age distribution, it remains a major contributor to morbidity and mortality in old age.^30^ There is extensive evidence that HIV infection could cause cognitive impairment and dementia, so some dementia cases reported here could have been linked to HIV. Surprisingly, preliminary studies from HAALSI showed that people living with HIV performed as well as or better than individuals who were HIV-negative on some common cognitive tests.^31^ Many possible explanations could account for this observation (eg, selection bias or increased health system interaction), and longitudinal monitoring paired with neuroimaging will be needed to fully unpack the relationship between HIV and dementia.

Our study has several strengths. It incorporates consensus diagnoses and algorithmic modelling to estimate individual-level and population-level dementia probability within a challenging research setting. We leverage an innovate web-based platform to facilitate timely collection of consensus diagnoses. Our study shows high concordance between two approaches for estimating dementia prevalence and consistent performance in the cross-validated datasets, providing confidence in the reliability of results. Our findings are based on a population-based sample that is generalisable to other rural communities in South Africa. Notably, our use of harmonised assessments and prediction models is designed to enable cross-national and cross-cohort comparisons of dementia in a way that accommodates heterogeneity in language, culture, and education. With proper calibration of cognitive tests, dementia algorithms can be developed to generate comparable dementia outcomes and facilitate a better understanding of risk factor patterns across countries.^28^

Nevertheless, acknowledging limitations is important. Our sample is not a nationally representative cohort, and findings might not generalise to urban areas in South Africa. However, we have generated insights into the burden of dementia in rural regions of sub-Saharan Africa, which could be disproportionately affected by dementia in the upcoming decades. Expansion of HAALSI–HCAP to a South African national survey is anticipated in 202G. Respondents who required a proxy during HAALSI Wave 2 were not included in the primary algorithms; however, sensitivity analyses showed no degradation in model performance when proxies were included. Additionally, individuals classified as mild cognitive impairment by the expert panel were grouped with cognitively unimpaired respondents for analytical purposes. Although we recognise that mild cognitive impairment could encompass individuals on the precipice of dementia, we have focused on identifying respondents with observable cognitive and functional impairment, as these data are most meaningful to inform social policy.

The projected increase in the number of people living with dementia will strain economic and social structures of ageing societies,^4^ particularly in low-income and middle-income countries.^5^ It is imperative to have higher quality epidemiological data on cognitive function and dementia throughout the sub-Saharan African region. Harmonising dementia assessments across studies will enable a better understanding of the multifactorial causes of dementia, while providing insight into where resources are most urgently needed.

## Supplementary Material

1

2

## Figures and Tables

**Table 1:** Descriptive characteristics of HAALSI Wave 2 and HAALSI–HCAP Wave 1 self-respondents

	HAALSI Wave 2	HAALSI−HCAP
	(N=3440)	Wave 1 (N=615)

**Demographics**		
Sex		
Female	1926 (56·0%)	377 (61·3%)
Male	1514 (44·0%)	238 (38·7%)
Age, years	66 (58−75)	69 (59−78)
Age group, years		
50−59	1010 (29·4%)	157 (25·5%)
60−69	1122 (32·6%)	166 (27·0%)
70−79	810 (23·5%)	160 (26·0%)
≥80	498 (14·5%)	132 (21·5%)
Years of schooling	3·3 (4·2)	2·7 (3·8)
Education		
No education	1612 (46·9%)	338 (55·0%)
Some primary	1248 (36·3%)	207 (33·7%)
Secondary or higher	571 (16·6%)	69 (11·2%)
Marital status		
Never married	197 (5·7%)	34 (5·5%)
Separated or divorced	400 (11·6%)	68 (11·1%)
Widowed	1277 (37·1%)	266 (43·3%)
Currently married or living apart	1558 (45·3%)	247 (40·2%)
Wealth index		
Quintile 1 (lowest)	695 (20·2%)	146 (23·7%)
Quintile 2	673 (19·6%)	121 (19·7%)
Quintile 3	650 (18·9%)	122 (19·8%)
Quintile 4	693 (20·1%)	114 (18·5%)
Quintile 5 (highest)	729 (21·2%)	112 (18·2%)
**Outcomes**		
Immediate recall score, 0−30	15·8 (5·1)	14·4 (5·3)
Delayed recall score, 0−10	4·8 (2·1)	4·3 (2·2)
Orientation score, 0−4	3·0 (1·5)	2·6 (1·6)
Self-reported memory, fair or poor	0·2 (0·4)	0·3 (0·4)
Verbal fluency score, 0−14	11·8 (3·9)	11·7 (3·8)
Sum score of days of the week,	13·1 (2·2)	12·8 (2·5)
0−14		

Data are n (%), mean (SD), or median (IQR). HAALSI=Health and Aging in Africa: A Longitudinal Study in South Africa. HAALSI−HCAP=HAALSI Dementia−Harmonized Cognitive Assessment Protocol.

**Table 2:** Weighted prevalence of dementia by age group at Health and Aging in Africa: A Longitudinal Study in South Africa Wave 2

	Full sample including proxy respondents	Full sample excluding proxy respondents
	(95% CI)	(95% CI)

Total	18% (15−22)	15% (13−18)
Age group, years		
50−59	5% (2−11)	4% (2−9)
60−69	12% (7−18)	10% (6−15)
70−79	25% (19−33)	23% (17−31)
≥80	42% (33−52)	36% (28−45)

**Table 3:** Model performance parameters at selected probability cut points

	Sensitivity, % (95% CI)	Specificity, % (95% CI)	Accuracy, % (95% CI)	Area under receiver operating characteristic curve (95% CI)	Youden’s J statistic	Selected cut point

**Model 1**						
Total	71·2% (63·3−79·1)	75·1% (71·3−78·9)	74·3% (70·8−77·8)	0·78 (0·74−0·83)	0·46	0·22
Sex						
Male	52·3% (37·5−67·1)	80·9% (75·4−86·4)	75·6% (70·1−81·1)	0·75 (0·68−0·83)	··	··
Female	81·5% (73·0−90·0)	71·3% (66·1−76·5)	73·5% (69·0−78·0)	0·81 (0·75−0·87)	··	··
Age group, years						
≥70	78·8% (70·7−86·9)	57·5% (50·5−64·5)	64·7% (59·2−70·2)	0·73 (0·66−0·79)	··	··
50−69	42·3% (23·3−61·3)	86·5% (82·6−90·4)	83·0% (78·9−87·1)	0·73 (0·63−0·83)	··	··
Education groups						
No formal education	77·5% (69·4−85·6)	58·9% (52·6−65·2)	64·5% (59·4−69·6)	0·73 (0·67−0·79)	··	··
Some education	45·5% (24·7−66·3)	90·2% (86·5−93·9)	86·6% (82·6−90·6)	0·75 (0·62− 0·87)	··	··
**Model 2**						
Total	70·4% (62·4−78·4)	81·0% (77·5−84·5)	78·9% (75·7−82·1)	0·82 (0·78−0·86)	0·51	0·29
Sex						
Male	56·8% (42·2−71·4)	83·5% (78·3−88·7)	78·6% (73·4−83·8)	0·78 (0·71− 0·85)	··	··
Female	77·8% (68·7−86·9)	79·4% (74·8−84·0)	79·0% (74·9−83·1)	0·84 (0·79−0·89)	··	··
Age groups, years						
≥70	81·8% (74·2−89·4)	53·9% (46·9−60·9)	63·4% (57·9−68·9)	0·73 (0·67−0·79)	··	··
50−69	26·9% (9·9−43·9)	98·7% (97·4−100·0)	92·9% (90·1−95·7)	0·76 (0·66−0·86)	··	··
Education groups						
No formal education	75·5% (67·2−83·8)	68·6% (62·7−74·5)	70·7% (65·8−75·6)	0·76 (0·71−0·82)	··	··
Some formal education	50·0% (29·1−70·9)	92·5% (89·3−95·7)	89·1% (85·4−92·8)	0·80 (0·70−0·90)	··	··
**Model 3**						
Total	71·5% (63·5−79·5)	81·1% (77·6−84·6)	79·2% (76·0−82·4)	0·83 (0·79−0·87)	0·53	0·29
Sex						
Male	58·1% (43·4−72·8)	81·4% (75·9−86·9)	77·2% (71·9−82·5)	0·79 (0·72−0·86)	··	··
Female	78·8% (69·8−87·8)	81·0% (76·5−85·5)	80·5% (76·5−84·5)	0·85 (0·81−0·90)	··	··
Age groups, years						
≥70	83·8% (76·5−91·1)	55·7% (48·7−62·7)	65·3% (59·8−70·8)	0·75 (0·69−0·81)	··	··
50−69	20·8% (4·6−37·0)	97·6% (95·9−99·3)	91·9% (88·9−94·9)	0·78 (0·68−0·88)	··	··
Education groups						
No formal education	78·2% (70·1−86·3)	67·5% (61·5−73·5)	70·7% (65·8−75·6)	0·78 (0·72−0·83)	··	··
Some formal education	40·9% (20·4−61·4)	93·7% (90·7−96·7)	89·5% (85·9−93·1)	0·81 (0·72−0·90)	··	··
**Model 4**						
Total	70·7% (62·7−78·7)	83·2% (79·9−86·5)	80·7% (77·6−83·8)	0·84 (0·80−0·88)	0·54	0·25
Sex						
Male	53·5% (38·6−68·4)	83·5% (78·3−88·7)	78·1% (72·8−83·4)	0·79 (0·73−0·86)	··	··
Female	80·0% (71·2−88·8)	83·0% (78·7−87·3)	82·4% (78·5−86·3)	0·86 (0·81−0·91)	··	··
Age groups, years						
≥70	79·8% (71·9−87·7)	61·5% (54·6−68·4)	67·7% (62·3−73·1)	0·77 (0·71−0·83)	··	··
50−69	33·3% (14·4−52·2)	97·3% (95·5−99·1)	92·5% (89·6−95·4)	0·80 (0·70−0·89)	··	··
Education groups						
No formal education	77·2% (69·0−85·4)	70·9% (65·1−76·7)	72·8% (68·0−77·6)	0·79 (0·74−0·84)	··	··
Some formal education	40·9% (20·4−61·4)	94·5% (91·7−97·3)	90·2% (86·7−93·7)	0·80 (0·71−0·90)	··	··

Sensitivity refers to correct detection of true dementia cases; specificity refers to correct detection of non-cases.

## Research in context

### Evidence before this study

On July 20, 2023, we searched PubMed and Google Scholar from database inception through to July, 2023, to identify articles reporting dementia prevalence and incidence rates from sub-Saharan African countries. Search terms used were (((dementia[Title/Abstract]) OR (alzheimer[Title/Abstract])) AND (prevalence[Title/Abstract])) AND (Africa[Title/Abstract]). We also referred to recently published review papers examining dementia prevalence and risk factors in sub-Saharan Africa and regional dementia prevalence estimates reported in the 2019 Global Burden of Disease Study. Published dementia prevalence estimates from sub-Saharan African countries ranged dramatically from 2% to 22%, likely stemming from variability in sampling, settings, and diagnostic methods.

### Added value of this study

The Health and Aging in Africa: A Longitudinal Study of an INDEPTH Community in South Africa (HAALSI) study provides robust longitudinal data on cognition, everyday function, and biological indicators of dementia. In this Article, we combine gold-standard consensus diagnoses and dementia prediction algorithms to provide urgently needed data on the prevalence of dementia in a region of the world undergoing rapid population ageing. Although algorithmic estimation of dementia prevalence has been successfully implemented in the USA and other high-income countries, HAALSI is among the first to combine consensus diagnoses with prediction algorithms to estimate dementia in the African context. This approach is especially valuable in resource-constrained settings in which comprehensive diagnostic tests on large samples might not be possible due to limited funding, shortage of qualified personnel, and poor health-care infrastructure.

### Implications of all the available evidence

Sub-Saharan Africa, and South Africa specifically, is projected to experience a dramatic increase in dementia prevalence by 2050. In 2024, the population of adults aged 60 years and older was estimated at 6·1 million. Although dementia prevalence is likely to vary across rural and urban areas, our data suggest that more than one million South Africans could be living with dementia in the upcoming decades. This study solidifies our understanding of the high burden of dementia in rural South Africa and provides insight into where resources are most urgently needed. Future research can use these approaches to model factors that influence dementia incidence and progression.

## References

[1] Livingston G, Huntley J, Sommerlad A, Dementia prevention, intervention, and care: 2020 report of the Lancet Commission. Lancet 2020; 396: 413–46. 32738937 10.1016/S0140-6736(20)30367-6 PMC7392084

[2] Livingston G, Sommerlad A, Orgeta V, Dementia prevention, intervention, and care. Lancet 2017; 390: 2673–734. 28735855 10.1016/S0140-6736(17)31363-6

[3] Wetterberg H, Najar J, Rydberg Sterner T, Decreasing incidence and prevalence of dementia among octogenarians: a population-based study on 3 cohorts born 30 years apart. J Gerontol A Biol Sci Med Sci 2023; 78: 1069–77. 36843145 10.1093/gerona/glad071 PMC10235204

[4] Johnsen B, Martinaityte I, Wilsgaard T, Schirmer H. Incidence of dementia over a period of 20 years in a Norwegian population. Alzheimers Dement (Amst) 2023; 15: e12479. 10.1002/dad2.12479 PMC10540268 37780861

[5] Prince M, Ali G-C, Guerchet M, Prina AM, Albanese E, Wu Y-T. Recent global trends in the prevalence and incidence of dementia, and survival with dementia. Alzheimers Res Ther 2016; 8: 23. 27473681 10.1186/s13195-016-0188-8 PMC4967299

[6] Gómez-Olivé FX, Montana L, Wagner RG, Cohort profile: Health and Ageing in Africa: a Longitudinal Study of an INDEPTH community in South Africa (HAALSI). Int J Epidemiol 2018; 47: 689–90j. 29325152 10.1093/ije/dyx247 PMC6005147

[7] UN. World population ageing 2019: highlights. 2019. https://www.un.org/en/development/desa/population/publications/pdf/ageing/WorldPopulationAgeing2019-Highlights.pdf (accessed Sept 20, 2024).

[8] Akinrolie O, Iwuagwu AO, Kalu ME, Longitudinal studies of aging in sub-Saharan Africa: review, limitations, and recommendations in preparation of projected aging population. Innov Aging 2024; 8: igae002. 10.1093/geroni/igae002 PMC11020233 38628825

[9] Mukadam N, Sommerlad A, Huntley J, Livingston G. Population attributable fractions for risk factors for dementia in low-income and middle-income countries: an analysis using cross-sectional survey data. Lancet Glob Health 2019; 7: e596–603. 31000129 10.1016/S2214-109X(19)30074-9 PMC7617123

[10] Akinyemi RO, Yaria J, Ojagbemi A, Dementia in Africa: current evidence, knowledge gaps, and future directions. Alzheimers Dement 2022; 18: 790–809. 34569714 10.1002/alz.12432 PMC8957626

[11] Ojagbemi A, Okekunle AP, Babatunde O. Dominant and modifiable risk factors for dementia in sub-Saharan Africa: a systematic review and meta-analysis. Front Neurol 2021; 12: 627761. 10.3389/fneur.2021.627761 PMC8027065 33841302

[12] Ranson JM, Kuźma E, Hamilton W, Muniz-Terrera G, Langa KM, Llewellyn DJ. Predictors of dementia misclassification when using brief cognitive assessments. Neurol Clin Pract 2019; 9: 109–17. 31041124 10.1212/CPJ.0000000000000566 PMC6461420

[13] Bassil DT, Farrell MT, Wagner RG, Cohort profile update: cognition and dementia in the Health and Aging in Africa Longitudinal Study of an INDEPTH community in South Africa (HAALSI dementia). Int J Epidemiol 2022; 51: e217–26. 34871405 10.1093/ije/dyab250 PMC9365629

[14] Juster FT, Suzman R. An overview of the Health and Retirement Study. J Hum Resour 1995; 30: S7–56.

[15] Kahn K, Collinson MA, Gómez-Olivé FX, Profile: Agincourt health and socio-demographic surveillance system. Int J Epidemiol 2012; 41: 988–1001. 22933647 10.1093/ije/dys115 PMC3429877

[16] Collinson MA, Mudzana T, Mutevedzi T, Cohort profile: South African Population Research Infrastructure Network (SAPRIN). Int J Epidemiol 2022; 51: e206–16. 34966919 10.1093/ije/dyab261 PMC9365637

[17] Langa KM, Plassman BL, Wallace RB, The Aging, Demographics, and Memory Study: study design and methods. Neuroepidemiology 2005; 25: 181–91. 16103729 10.1159/000087448

[18] Little TD, Rhemtulla M. Planned missing data designs for developmental researchers. Child Dev Perspect 2013; 7: 199–204.

[19] Bassil DT, Farrell MT, Weerman A, Feasibility of an online consensus approach for the diagnosis of cognitive impairment and dementia in rural South Africa. Alzheimers Dement (Amst) 2023; 15: e12420. 10.1002/dad2.12420 PMC10072202 37025188

[20] Kobayashi LC, Farrell MT, Langa KM, Mahlalela N, Wagner RG, Berkman LF. Incidence of cognitive impairment during aging in rural South Africa: evidence from HAALSI, 2014 to 2019. Neuroepidemiology 2021; 55: 100–08. 33657567 10.1159/000513276 PMC8058235

[21] Farrell MT, Kobayashi LC, Montana L, Wagner RG, Demeyere N, Berkman L. Disparity in educational attainment partially explains cognitive gender differences in older rural South Africans. J Gerontol B Psychol Sci Soc Sci 2020; 75: e161–73. 32211786 10.1093/geronb/gbaa035 PMC7424269

[22] McKhann GM, Knopman DS, Chertkow H, The diagnosis of dementia due to Alzheimer’s disease: recommendations from the National Institute on Aging-Alzheimer’s Association workgroups on diagnostic guidelines for Alzheimer’s disease. Alzheimers Dement 2011; 7: 263–69. 21514250 10.1016/j.jalz.2011.03.005 PMC3312024

[23] Ojagbemi A, Bello T, Gureje O. Cognitive reserve, incident dementia, and associated mortality in the Ibadan Study of Ageing. J Am Geriatr Soc 2016; 64: 590–95. 26926137 10.1111/jgs.14015 PMC4976799

[24] Uwakwe R, Ibeh CC, Modebe AI, The epidemiology of dependence in older people in Nigeria: prevalence, determinants, informal care, and health service utilization. A 10/66 dementia research group cross-sectional survey. J Am Geriatr Soc 2009; 57: 1620–27. 19682135 10.1111/j.1532-5415.2009.02397.x

[25] Paddick S-M, Longdon AR, Kisoli A, Dementia prevalence estimates in sub-Saharan Africa: comparison of two diagnostic criteria. Glob Health Action 2013; 6: 19646. 23561025 10.3402/gha.v6i0.19646 PMC3617645

[26] Prince M, Acosta D, Chiu H, Scazufca M, Varghese M. Dementia diagnosis in developing countries: a cross-cultural validation study. Lancet 2003; 361: 909–17. 12648969 10.1016/S0140-6736(03)12772-9

[27] Hall KS, Gao S, Emsley CL, Ogunniyi AO, Morgan O, Hendrie HC. Community screening interview for dementia (CSI ‘D’); performance in five disparate study sites. Int J Geriatr Psychiatry 2000; 15: 521–31. 10861918 10.1002/1099-1166(200006)15:6<521::aid-gps182>3.0.co;2-f

[28] Vonk JMJ, Gross AL, Zammit AR, Cross-national harmonization of cognitive measures across HRS HCAP (USA) and LASI-DAD (India). PLoS One 2022; 17: e0264166. 10.1371/journal.pone.0264166 PMC8880818 35213581

[29] Gianattasio KZ, Wu Q, Glymour MM, Power MC. Comparison of methods for algorithmic classification of dementia status in the health and retirement study. Epidemiology 2019; 30: 291–302. 30461528 10.1097/EDE.0000000000000945 PMC6369894

[30] Rosenberg MS, Gómez-Olivé FX, Rohr JK, Sexual behaviors and HIV status: a population-based study among older adults in rural South Africa. J Acquir Immune Defic Syndr 2017; 74: e9–17. 27926667 10.1097/QAI.0000000000001173 PMC5147032

[31] Asiimwe SB, Farrell M, Kobayashi LC, Cognitive differences associated with HIV serostatus and antiretroviral therapy use in a population-based sample of older adults in South Africa. Sci Rep 2020; 10: 16625. 33024208 10.1038/s41598-020-73689-7 PMC7539005

