# Supplementary material for: Estimating dementia prevalence using remote diagnoses and algorithmic modelling: a population-based study of a rural region in South Africa
Source: Lancet Glob Health. Author manuscript; Available in PMC 2025 Apr 11. (PMC11987161; doi:10.1016/S2214-109X(24)00325-5)
Supplement: 1 [file NIHMS2037430-supplement-1.pdf]

THE LANCET
Global Health

Supplementary appendix 1

This appendix formed part of the original submission and has been peer reviewed. We post it as supplied by the authors.

Supplement to: Farrell MT, Bassil DT, Guo M, et al. Estimating dementia prevalence using remote diagnoses and algorithmic modelling: a population-based study of a rural region in South Africa. *Lancet Glob Health* 2024; **12:** e2003–11.

Estimating dementia prevalence using remote diagnoses and algorithmic modelling: a population-based study of a rural region in South Africa

Supplemental Materials

Contents

Sampling 2

Supplemental Figure 1. Flow chart depicting sampling strategy and response rates in the HAALSI-HCAP Study 3

Brief Description of Data Collection Methods 4

Statistical Analyses 5

Supplemental Figure 2. Prevalence of dementia by age and education groups across prediction methods 6

Supplemental Table 1: Predictors included in each model 7

Supplemental Table 2. Coefficients from models obtained in the full HAALSI-HCAP dataset ... 8

Supplemental Table 3. Model performance metrics for the fivefold cross-validated datasets 9

Supplemental Table 4. Model performance metrics when estimating dementia in HAALSI-HCAP with proxy respondents included in models 10

Supplemental Table 5. Sensitivity analyses demonstrating model performance parameters for models based on full set of cognitive predictors (Model 5, n=520) 11

Sampling

Detailed Description of Sampling Procedures

The HAALSI-HCAP cohort was drawn from 4,176 respondents who completed the second wave (2018–2019) of HAALSI. The sample was restricted to those aged 50 years and older. Respondents who completed the HAALSI survey independently were referred to as self-respondents (n=3,932), and those who required a proxy to complete the survey were referred to proxy respondents (n=244). After excluding self-respondents with missing cognitive data (i.e., missing immediate recall or orientation scores), there were 3,440 self-respondents ≥ 50 years eligible for inclusion in the HAALSI-HCAP study. Based on cognitive scores at HAALSI Wave 2, self-respondents were stratified into four dementia risk levels: Highest-, High-, Low- and Lowest-Risk (Supplemental Figure 1). Dementia risk categories used for sampling were determined by examining associations between cognitive screening scores and dementia diagnoses in a prior pilot study. Among eligible self-respondents, 42.81% were in the Lowest-Risk group, 35.63% were in the Low-Risk group, 16.03% were in the High-Risk group, and 5.53% were in the Highest-Risk group. Using simulated data, an optimal sampling strategy was developed for the HAALSI-HCAP sub-sample to maximize precision in estimating dementia prevalence in the parent cohort, consisting of 30% each from the Lowest-, Low-, and High-Risk groups and 10% from the Highest-risk group. We estimated a sample size of 600 respondents would achieve a 95% CI of +/- 0.05 for estimated dementia prevalence in HAALSI.

In total, 690 HAALSI participants were invited to take part in the HAALSI HCAP study, including a random sample of 207 individuals (30%) each from the Lowest-, Low- and High-risk groups, and 69 (10%) from the Highest Risk group. Additionally, 20 participants who required a proxy in HAALSI Wave 2 were recruited, assuming high likelihood of dementia among these participants. Of the 690 respondents invited, 632 completed the HAALSI-HCAP assessment (91.6% response rate). The distribution of the final HAALSI-HCAP sample included 181 individuals (28.64%) from the lowest-risk stratum, 188 (29.75%) from low-risk stratum, 182 (28.8%) from high-risk stratum, and 64 (10.13%) from the highest-risk stratum, reflecting the intentional oversampling of higher-risk respondents. Additionally, 17 (2.68%) participants who required a proxy in HAALSI wave 2 were recruited into the HAALSI-HCAP sub-study. There was a high overall response rate which did not differ across age, sex, or dementia risk stratum (Supplemental Figure 1).

Development of Sampling Weights

Sampling weights were constructed to allow us to reweight the HAALSI-HCAP sub-cohort back to the parent HAALSI population. Each person's weight reflects their inverse probability of participating in the HAALSI-HCAP study given their assigned risk stratum. First, for self-respondents, we calculated the probability of being sampled into each risk stratum (number of respondents sampled within risk group/ total HAALSI respondents within risk group). Next, we calculated the probability of HAALSI-HCAP completion (number of HAALSI-HCAP completers in risk group/ number of participants sampled in risk group). Finally, we calculated each respondent's probability of taking part in HAALSI-HCAP by multiplying their probability of being sampled by their probability of completing the survey. The final weight is the reciprocal of this probability value. The same approach was followed for respondents requiring a proxy. We included proxy respondents aged 50 and over (n=222) and randomly selected 20 of them. Next, we multiplied the probability of being sampled (number of proxy respondents sampled / total

number of HAALSI respondents requiring proxy) by the probability of completion (number of proxy respondent completers/ number of proxy respondents sampled), and generated weights that reflect the inverse of this probability value.

Supplemental Figure 1. Flow chart depicting sampling strategy and response rates in the HAALSI-HCAP Study

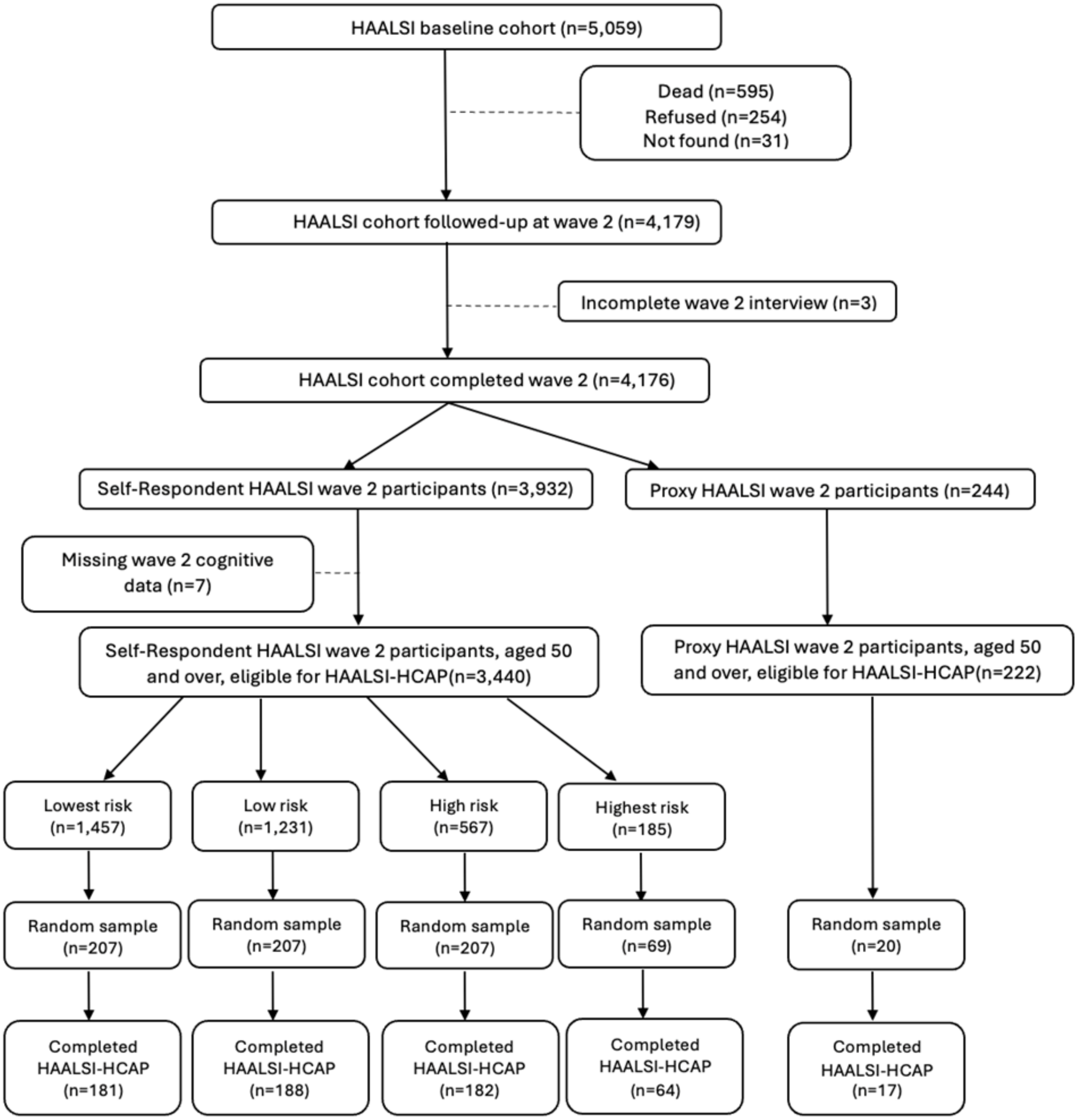

Brief Description of Data Collection Methods

The data collection team, consisting of local nurses and fieldworkers, underwent extensive training covering the study protocol, consent procedures, and data management. Training sessions were led by research team specialists proficient in neuropsychological testing, geriatric care, and neurological examinations. All data were collected using the Computer-Assisted Personal Interviewing (CAPI) software on Samsung galaxy tablets to ensure simultaneous data capture. Weekly data quality checks were conducted to promptly address any data discrepancies, including potential errors in fieldworkers' administration of cognitive measures.

Interviews and clinical assessments occurred at participants' homes. In instances where cognitive impairment hindered direct participation in cognitive testing, proxy consent was sought from a designated family member authorized to make decisions on the participant's behalf. Nevertheless, informant interviews and, when feasible, neurological examinations were still administered for these individuals. Participants with limited literacy provided verbal consent in the presence of a witness.

For further details on the methodology and the cohort, please refer to our cohort profile paper on the HAALSI-Dementia Study (https://pubmed.ncbi.nlm.nih.gov/34871405/)

Statistical Analyses

Equations for logistic regression algorithms

$$\log\left(\frac{P}{1-P}\right) = \alpha_0 + \beta X + \varepsilon$$

P: probability of having dementia
β: a set of estimates of predictors
X: dementia predictors
ε: random error

Cross-validation procedure

To evaluate the predictive performance of each algorithm, we employed a 5-fold cross-validation approach, accounting for our modest sample size of 615 respondents. First, we randomly split the sample into five folds. Next, we applied logistic regression models to predict dementia; models were trained on data from four folds and used to generate predicted probabilities of dementia for respondents in the remaining fold. This process was repeated five times, ensuring that each fold served as the validation set exactly once. Within each fold, dementia probability cut points were generated 0.01 to 0.99 with a 0.01 increment. Algorithm-based dementia statuses were established so that predicted probabilities equal to or exceeding the specified cut point were classified as having dementia. We compared the algorithm-based dementia statuses against the diagnoses obtained with consensus procedures, calculating sensitivity, specificity, and accuracy for each cut point. We averaged the performance metrics across all five folds, providing an average assessment of algorithmic effectiveness. Finally, at each cut point, we calculated Youden's J statistics (sensitivity + specificity – 1).

Bootstrapping procedure

To assess the uncertainty level of algorithm performance metrics, we implemented a bootstrapping approach. This involved repeating the cross-validation procedure 100 times, with each iteration based on a resampled HCAP sample. Each resample was drawn with replacement and maintained the same size as the original HCAP sample. We selected values at 2.5th percentile and 97.5th percentile respectively as lower and upper level of a certain performance metric's 95% confidence interval.

Supplemental Figure 2. Prevalence of dementia by age and education groups across prediction methods

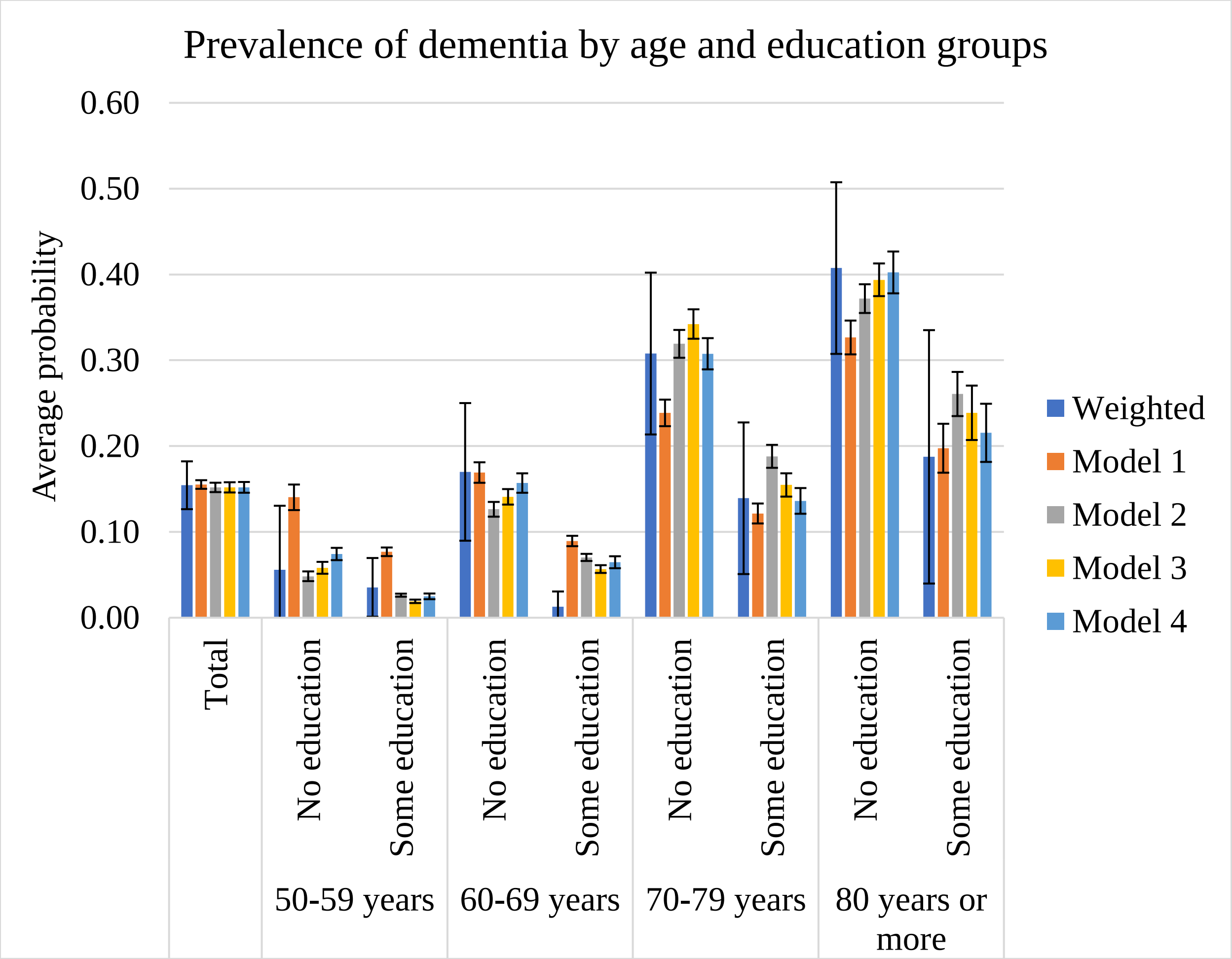

Notes. Weighted estimates were based on HCAP sample (n=615). Model estimates were obtained by applying each model to HAALSI wave 2 respondents aged 50 or over (n=3440). Error bars represent the 95% confidence intervals of the point estimates assuming a normal distribution.

Supplemental Table 1: Predictors included in each model

Predictors	Model 1	Model 2	Model 3	Model 4	Model 5
Immediate recall	X	X	X	X	X
Immediate recall * ADL				X	
Delayed recall	X	X	X		X
Orientation score	X	X	X		X
Orientation * ADL				X	
Orientation * iADL				X	
Orientation * Education				X	
Orientation * Female				X	
Self-reported memory	X	X	X		X
Self-reported memory * ADL				X	
Self-reported memory * age				X	
Self-reported memory * female				X	
Verbal fluency			X	X	X
Verbal fluency * iADL				X	
Sum score of days of the week			X		X
Incidental memory					X
Picture naming					X
Trail making					X
Number series					X
ADLs	X	X	X		X
IADLs			X		X
Age		X	X	X	X
Age * Age 80+		X	X	X	X
Education			X		X
Female			X		X

Notes: X means the variable was included in the prediction model. ADL: Activities of Daily Living; IADL: Instrumental Activities of Daily living
Model 4: Predictors selected through least absolute shrinkage and selection operator (LASSO)
Model 5: Includes cognitive measures with significant missingness (> 5%)

Supplemental Table 2. Coefficients from models obtained in the full HAALSI-HCAP dataset

	Model 1 (N=615)		Model 2 (N=615)		Model 3 (N=611)		Model 4 (N=611)		Model 5 (N=520)	
Predictors	Coefficient	p value	Coefficient	p value	Coefficient	p value	Coefficient	p value	Coefficient	p value
Self-respondent cognitive measures										
Immediate recall	-0.081	*	-0.056		-0.046		-0.083	**	-0.059	
Delayed recall	-0.050		-0.078		-0.050				0.016	
Orientation score	-0.421	***	-0.328	***	-0.239	**			-0.159	
Self-reported memory (fair or poor)	0.420		0.257		0.243				0.397	
Verbal fluency					-0.064		-0.081		-0.052	
Sum score of days of the week					0.011				0.074	
Incidental memory									-0.066	
Adaptive number series									-0.001	
Trail making									-0.018	
Picture naming									-0.130	*
Function										
ADLs	0.125		0.081		-0.190				-0.202	
IADLs					0.221	**			0.196	*
Socio-demographic variables										
Age			0.096	***	0.091	***	0.054	**	0.087	***
Age≥ 80 years			10.317	**	11.140	**			11.294	*
Education					-0.103				-0.088	
Female					-0.057				0.142	
Interactions										
Age * oldest (age>=80 years)			-0.134	**	-0.145	**	0.008		-0.148	**
Immediate recall * ADL							0.084	*		
Orientation * ADL							-0.281			
Orientation * iADL							-0.084			
Orientation * Education							-0.039	*		
Orientation * Female							-0.156			
Self-reported memory * ADL							-1.393	**		
Self-reported memory * age							0.004			
Self-reported memory * female							0.380			
Verbal fluency * iADL							0.036			
Intercept	0.631		-6.415	***	-5.894	**	-3.737	*	-5.074	*

Notes: *** p<0.001; ** p <0.01, * p<0.05. Models were based on full HCAP sample.

Supplemental Table 3. Model performance metrics for the fivefold cross-validated datasets

	Sensitivity % (95% CI)	Specificity % (95% CI)	Accuracy % (95% CI)	Cut-point
**Model 1**				
Total	68.5 (58.3-76.6)	75.3 (68.8-81.2)	74.1 (68.4-79.3)	0.22
Gender				
Male	49.2 (32.2-65.1)	81.1 (74.7-87.9)	75.5 (69.5-82.1)	
Female	78.9 (67.1-87.4)	71.7 (63.1-79.0)	72.9 (67.0-79.5)	
Age groups				
≥70	75.6 (62.2-85.1)	59.2 (48.4-70.2)	64.6 (58.2-71.8)	
50-70	43.2 (19.8-57.3)	85.5 (78.5-91.8)	82.2 (75.1-89.0)	
Education groups				
No formal education	74.3 (61.1-81.8)	59.5 (49.1-70.1)	63.8 (57.9-71.1)	
Some formal education	45.2 (20.6-71.7)	89.2 (82.9-93.8)	85.9 (78.5-90.5)	
**Model 2**				
Total	64.6 (50.3-74.3)	81.2 (76.9-86.3)	78.0 (74.1-81.6)	0.29
Gender				
Male	50.4 (28.4-71.5)	84.4 (76.4-90.5)	78.1 (72.3-83.9)	
Female	71.5 (53.0-82.2)	79.6 (73.9-85.6)	78.0 (72.0-82.0)	
Age groups				
≥70	75.0 (62.7-84.6)	56.8 (42.4-67.5)	62.9 (55.5-69.5)	
50-70	25.4 (4.3-48.3)	97.7 (95.1-99.5)	91.5 (87.5-95.8)	
Education groups				
No formal education	68.0 (54.8-79.6)	69.1 (61.5-78.0)	68.7 (63.5-74.7)	
Some formal education	46.3 (19.2-71.9)	92.9 (88.4-96.9)	88.9 (83.6-93.8)	
**Model 3**				
Total	63.7 (46.6-77.3)	81.9 (78.2-86.8)	78.3 (75.0-83.3)	0.29
Gender				
Male	50.5 (26.0-73.2)	83.0 (74.4-89.1)	77.1 (70.5-83.2)	
Female	71.4 (56.6-83.6)	81.8 (75.5-88.2)	79.6 (74.9-85.8)	
Age groups				
≥70	75.1 (58.1-86.5)	58.1 (50.3-70.4)	64.0 (57.7-71.1)	
50-70	24.2 (0.0-45.1)	97.0 (92.5-99.5)	91.4 (86.4-94.8)	
Education groups				
No formal education	70.2 (52.9-81.9)	68.7 (59.7-77.7)	69.0 (63.5-76.3)	
Some formal education	41.4 (11.4-63.7)	94.2 (90.3-97.6)	89.6 (84.3-94.9)	
**Model 4**				
Total	67.2 (56.5-76.2)	81.2 (76.0-86.9)	78.4 (73.7-82.7)	0.25
Gender				
Male	51.5 (29.0-70.1)	81.5 (71.5-88.6)	75.7 (66.8-82.2)	
Female	76.3 (63.6-86.2)	81.4 (73.9-87.7)	80.1 (73.9-85.3)	
Age groups				
≥70	76.2 (65.3-84.8)	59.1 (45.5-70.6)	64.8 (57.3-72.7)	
50-70	33.3 (8.1-57.8)	95.3 (91.2-98.3)	90.5 (85.1-94.1)	
Education groups				
No formal education	72.5 (60.5-82.2)	67.9 (58.8-78.2)	69.2 (61.9-76.3)	
Some formal education	46.3 (25.1-71.2)	93.4 (89.8-97.3)	89.6 (85.3-94.0)	

Supplemental Table 4. Model performance metrics when estimating dementia in HAALSI-HCAP with proxy respondents included in models

	Sensitivity % (95% CI)	Specificity % (95% CI)	Accuracy % (95% CI)	ROC area	Youden's J statistic	Cut-point
**Model 1**						
Total	73.3%	74.0%	73.9%	0.79	0.47	0.22
Gender						
Male	55.3%	79.2%	74.6%	0.76		
Female	83.0%	70.7%	73.5%	0.81		
Age groups						
≥70	80.2%	56.9%	65.1%	0.74		
50-70	48.3%	85.1%	81.9%	0.75		
Education groups						
No formal education	79.1%	57.3%	64.1%	0.73		
Some formal education	50.0%	89.8%	86.4%	0.76		
**Model 2**						
Total	74.1%	79.9%	78.6%	0.82	0.54	0.28
Gender						
Male	61.7%	82.7%	78.7%	0.79		
Female	80.7%	78.0%	78.6%	0.84		
Age groups						
≥70	84.9%	52.8%	64.1%	0.74		
50-70	34.5%	97.4%	91.8%	0.78		
Education groups						
No formal education	79.1%	67.2%	70.9%	0.77		
Some formal education	54.2%	91.8%	88.6%	0.81		
**Model 3**						
Total	75.2%	79.8%	78.8%	0.83	0.55	0.28
Gender						
Male	60.9%	80.7%	77.0%	0.79		
Female	82.8%	79.2%	80.0%	0.86		
Age groups						
≥70	86.8%	54.6%	66.0%	0.75		
50-70	29.6%	96.0%	90.5%	0.79		
Education groups						
No formal education	81.7%	65.7%	70.7%	0.78		
Some formal education	45.8%	93.0%	88.9%	0.82		
**Model 4**						
Total	72.2%	82.8%	80.6%	0.84	0.55	0.26
Gender						
Male	56.5%	83.2%	78.2%	0.80		
Female	80.5%	82.6%	82.1%	0.86		
Age groups						
≥70	80.2%	62.4%	68.7%	0.78		
50-70	40.7%	96.0%	91.5%	0.81		
Education groups						
No formal education	78.0%	71.1%	73.3%	0.79		
Some formal education	45.8%	93.8%	89.6%	0.82		

Supplemental Table 5. Sensitivity analyses demonstrating model performance parameters for models based on full set of cognitive predictors (Model 5, n=520)

	Sensitivity %(95% CI)	Specificity %(95% CI)	Accuracy %(95% CI)	ROC area	Youden's J statistic	Cut point
**Model 5**						
Total	72.0%	84.1%	81.9%	0.84	0.56	0.26
Gender						
Male	61.3%	84.3%	80.8%	0.78		
Female	77.4%	83.9%	82.6%	0.86		
Age groups						
≥70	82.2%	60.2%	67.1%	0.75		
50-70	35.0%	98.5%	94.1%	0.79		
Education groups						
No formal education	77.6%	72.6%	74.0%	0.80		
Some formal education	47.1%	93.9%	90.7%	0.78		

Notes: Model 5 includes all Model 3 variables plus days of the week, trail making, picture naming, and adaptive number series as predictors.
