# Supplementary material for: Estimating dementia prevalence using remote diagnoses and algorithmic modelling: a population-based study of a rural region in South Africa
Source: Lancet Glob Health. Author manuscript; Available in PMC 2025 Apr 11. (PMC11987161; doi:10.1016/S2214-109X(24)00325-5)
Supplement: 2 [file NIHMS2037430-supplement-2.pdf]

THE LANCET
Global Health

Supplementary appendix 2

This Equitable Partnership Declaration (EPD) was submitted by the authors, and we reproduce it as supplied. It has not been peer reviewed. *The Lancet*'s editorial processes have not been applied to the EPD.

Supplement to: Farrell MT, Bassil DT, Guo M, et al. Estimating dementia prevalence using remote diagnoses and algorithmic modelling: a population-based study of a rural region in South Africa. *Lancet Glob Health* 2024; **12:** e2003–11.

Equitable Partnership Declaration questions

This Equitable Partnership Declaration is a statement being published online alongside papers at *The Lancet Global Health*, as a separate appendix, to allow researchers to describe how their work engages with researchers, communities, and environments in the countries of study. This is part of our broader goal to decolonise global health, handing control and leadership of research to academics and clinicians who are based in the regions of study, and to affected communities.

Please answer all questions with as much detail as possible, noting that all included information will be published open-access and it will be freely available online to all who wish to read it. If a question does not apply to your study, please state "Not applicable".

The format of and questions in this statement are currently in a pilot phase. Please email Dr Liam Messin (Liam.Messin@lancet.com; deputy editor) and Dr Kate McIntosh (Kate.McIntosh@lancet.com; senior editor) with any feedback, particularly if you find any questions unclear.

Researcher considerations

1. Please detail the involvement that researchers who are based in the region(s) of study had during a) study design; b) clinical study processes, such as processing blood samples, prescribing medication, or patient recruitment; c) data interpretation; and d) manuscript preparation, commenting on all aspects. If they were not involved in any of these aspects, please explain why.

This question is intended for international partnerships; if all your authors are based in the area of study, this question is not applicable.

This should include a thorough description of their leadership role(s) in the study. Are local researchers named in the author list or the acknowledgements, or are they not mentioned at all (and, if not, why)? Please also describe the involvement of early career researchers based in the location of the study. Some of this information might be repeated from the Contributors section in the manuscript. Note: we adhere to ICMJE authorship criteria when deciding who should be named on a paper.

Study design: The HAALSI Dementia Study is collaborative effort between academic researchers and clinicians based in South Africa (University of the Witwatersrand or "Wits" and South African Medical Research Council) and the United States (Harvard University, Columbia University, University of Michigan, and other collaborating institutions). The study co-principal investigators are based at Harvard Chan School of Public Health and the Faculty of Health Sciences, University of the Witwatersrand. Several co-authors on this manuscript were instrumental in writing the grant which funded the HAALSI Dementia Study. Collaborators at the University of the Witwatersrand, including graduate and post-doctoral level researchers, provided invaluable input on the study procedures, sampling methods, and outcome measures, and led participant recruitment and data collection. Community engagement was integral from the start.

HAALSI would not exist without the forward-thinking leadership of HAALSI co-PIs, one of whom – from Wits University in South Africa – founded the SAMRC/Wits-Agincourt health and socio-demographic surveillance system (HDSS). For over three decades, investigators and field staff have leveraged this population-based data collection platform to facilitate research and clinical training.

Since 1992, the Agincourt HDSS has generated critical insight into rural public health and population dynamics in rural northeast South Africa. The Agincourt HDSS provides the foundation for the Rural Public Health and Health Transitions Research Unit of the South African Medical Research Council (SAMRC) and University of the Witwatersrand, South Africa (the SAMRC/Wits-Agincourt Unit).
**b) Clinical study processes:** US- and South Africa-based clinicians (neurologists, neuropsychologists, and geriatricians) co-designed the neurological examination; researchers at all collaborating institutions provided input on the cognitive battery. The consensus diagnoses were assigned by a panel of clinical and research experts in South Africa and the United States. A magnetic resonance imaging (MRI) protocol was designed by a US-based neuroimaging expert (and study co-author) and tested and implemented by South African investigators based out of Wits and Kiaat Hospital in Nelspruit, South Africa. Data from the MRI study are not reported in the current manuscript.
**c) Data interpretation:** Statistical analyses were led by researchers at Harvard School of Public Health and Boston University. Results from this study have been presented at internal HAALSI meetings in the United States and South Africa, including meetings held in the Agincourt Field Research Centre.
**d) Manuscript preparation:** Wits co-authors have been engaged in the development of this manuscript since its inception and have provided critical input and feedback throughout the revision process.

2. Were the data used in your study collected by authors named on the paper, or have they been extracted from a source such as a national survey? ie, is this a secondary analysis of data that were not collected by the authors of this paper. If the authors of this paper were not involved in data collection, how were data interpreted with sufficient contextual knowledge?

   The Lancet Global Health *believe contextual understanding is crucial for informed data analysis and interpretation.*

Co-authors from Wits University led the community engagement, data collection and field-based quality checking effort for the HAALSI Dementia study. Successful data collection was made possible by the local HAALSI Fieldwork Manager based at the Agincourt HDSS research centre who helped to identify and address unexpected data patterns.

3. How was funding used to remunerate and enhance the skills of researchers and institutions based in the area(s) of study? And how was funding used to improve research infrastructure in the area of study?

   Potentially effective investments into long-term skills and opportunities within institutions could include training or mentorship in analytical techniques and manuscript writing, opportunities to lead all or specific aspects of the study, financial remuneration rather than requiring volunteers, and other professional development and educational opportunities.

Improvements to research infrastructure could be funding of extended trial designs (such as platform trials) and use of master protocols to enable these designs, establishment of long-term contracts for research staff, building research facilities, and local control of funding allocation.

**Skills:** Researchers and field staff at the SAMRC/Wits-Agincourt Unit are fulltime, grant funded positions. The HAALSI Dementia study supported up to 10 research positions based in the SAMRC/Wits-Agincourt Unit during the initial R01 funding period. During this time, field team members received extensive training in the implementation of digital cognitive tests (administration and scoring), as well as data interpretation. Agincourt team members in more advanced research roles (i.e., data managers, biostatisticians, project managers) participated in a data analysis and manuscript development workshop hosted at the Harvard TH Chan School of Public Health and the Harvard Center for Population and Development in Cambridge, Massachusetts. Since then, several manuscripts have been published by researchers from the SAMRC/Wits-Agincourt team. Beyond this, the Agincourt team were instrumental in initial curating of data and ensuring data integrity and anonymity prior to full team review.
**Research infrastructure:** The HAALSI Dementia R01 and HAALSI P01 grants (via the National Institute on Aging) have been used to purchase research equipment (e.g., tablets, laboratory equipment, audiometers), contribute to operational maintenance (e.g., generators, internet capabilities) and support salaries for research and field staff of the SAMRC/Wits-Agincourt Unit. All equipment purchased for HAALSI may help to mitigate costs of subsequent studies run through the SAMRC/Wits Agincourt unit. An important complementary contribution involved the financial and substantive contributions of the Dept of Science and Innovation (DSI), SAMRC leadership, and leaders of the SA Population Research Infrastructure Network (SAPRIN) in ensuring the overall integrity and continuity of the Agincourt HDSS – within which HAALSI Dementia is nested. ,

4. How did you safeguard the researchers who implemented the study?

Please describe how you guaranteed safe working conditions for study staff, including provision of appropriate personal protective equipment, protection from violence, and prevention of overworking.

The HAALSI Dementia Study procedures were reviewed and approved by local ethics committees through Wits University and the Mpumalanga province. Occupational Health and Safety in Agincourt and the field is well integrated in Wits and subject to periodic University review. Vehicles are provisioned by trained drivers to transport field staff and equipment during household data collection. All field staff take part in an extensive 4-week training programme to ensure that safety procedures are followed. Nevertheless, the realities of life and work in a rural, under-resourced environment brings unanticipated challenges including COVID-19, rapidly deteriorating road infrastructure, power outages and lack of potable water.

Benefits to the communities and regions of study

5. How does the study address the research and policy priorities of its location?

   How were the local priorities determined and then used to inform the research question? Who decided which priorities to take forward? Which elements of the study address those priorities?

The priorities for the HAALSI Dementia Study were guided by decades of epidemiological, demographic and social research from the Agincourt HDSS demonstrating shifting population dynamics related to health and social transitions. An increase in life expectancy in northeast South Africa, and sub-Saharan Africa more broadly, is projected to result in much higher proportions of individuals living to older age. With this demographic transition comes higher prevalence of age-related diseases, including dementia.

For the HAALSI Dementia Study, researchers at all collaborating institutions contributed to specific aims. Lower levels of educational attainment, high rates of HIV, and increasing levels of cardiometabolic diseases were identified as important risk factors for dementia that required further investigation.

The research objectives and procedures were further guided by input from the local Agincourt community. As with all studies run through the MRC/Wits-Agincourt Unit, the HAALSI Dementia Study began with a collaborative meeting between HAALSI researchers and our study-specific community advisory committee group (CAG). The CAG, composed of members of the Agincourt community, provided invaluable input on aspects of study design, including selection of culturally appropriate stimuli (e.g., photos for a picture naming task). The CAG also helped to contextualize potentially unexpected findings. For example, the CAG shed light on how persistent stigma around mental illness may affect community member's understanding of the causes and symptoms of dementia; these insights were important for determining how to incorporate informant/family member input into the dementia assessment.

This study has also inspired several other related projects aiming to understand unmet needs around dementia caregiving in the community, which we hope will lead to larger social policy improvements.

6. How will research products be shared in the community of study?

   For instance, will you be providing written or oral layperson summaries for non-academic information sharing? Will study data be made available to institutions in the region(s) of study? The Lancet Global Health *encourages authors to translate the summary (abstract) into relevant languages after paper editing; do you intend to translate your summary?*

In response to comments from the CAG, the HAALSI study provides its participants with individual-level results that are deemed to be clinically significant (e.g., abnormal MRI findings, blood pressure results). Participants are instructed on where to receive follow-up care.

Results from the HAALSI study have been shared regularly with the larger Agincourt and Bushbuckridge communities via flyers and information sheets. For this manuscript, we intend to translate the summary into the local language (Xitsonga), as well as Zulu to increase readership across South Africa. Of similar importance is ensuring that local clinic staff and district managers are properly informed of the implications of findings for primary care, clinical support and referral systems.

7. How were individuals, communities, and environments protected from harm?

a) *How did you ensure that sensitive patient data was handled safely and respectfully? Was there any potential for stigma or discrimination against participants arising from any of the procedures or outcomes of the study?*

Computer-Assisted Personal Interviewing (CAPI) software, built on encrypted Samsung galaxy tablets, was used for all assessments and simultaneous data capture. Rigorous procedures were implemented to ensure that confidentiality of participant data was maintained, including HIPAA compliant data storage and transfer systems. For data sharing, identifying information is removed from study data to protect anonymity. Data sharing via the Harvard Dataverse requires a very restrictive protocol for outside researchers to access sensitive data, including biomarker results. These efforts are paralleled by data held on the Agincourt Unit repository. Since 2014, there have been no breaches in confidentiality.

b) *Might any of the tests be experienced as invasive or culturally insensitive?*

No; the dementia diagnostic outcomes reported in this manuscript are based on informant report and cognitive testing that underwent extensive pilot testing to ensure cultural appropriateness.

c) *How did you determine that work was sensitive to traditions, restrictions, and considerations of all cultural and religious groups in the study population?*

All study questions and cognitive tests were extensively examined using qualitative and quantitative methods. Local advisors (field staff, CAB) reviewed the study stimuli to select the most relevant and appropriate items. Measures were pilot tested in the community to determine whether any items were considered problematic or produced unexpected data patterns with deliberate adjustment wherever indicated.

d) *Were biowaste and radioactive waste disposed of in accordance with local laws?*

Yes, all procedures (approved by Wits and Mpumalanga ethics committees) were meticulously followed.

e) *Were any structures built that would have impacted members of the community or the environment (such as handwashing facilities in a public space)? If so, how did you ensure that you had appropriate community buy-in?*

No, data collection occurred at participants' primary residence with full observance of consent procedures. Similarly, observance of local courtesies with household members as well as both traditional and civic structures were carefully followed, advised by the Agincourt Unit's Public Engagement team.

f) *How might the study have impacted existing health-care resources (such as staff workloads, use of equipment that is typically employed elsewhere, or reallocation of public funds)?*

Study procedures were conducted by trained fieldworkers and three local nurses. The study did not impact any local health facilities – apart from referrals to manage clinical risks and conditions.

8. Finally, please provide the title (eg, Dr/Prof, Mr/Mrs/Ms/Mx), name, and email address of an author who can be contacted about this statement. This can be the corresponding author.

Name: Prof Stephen Tollman
Email: Stephen.Tollman@wits.ac.za .
